# Feasibility of hybrid in-stream generator–photovoltaic systems for Amazonian off-grid communities

**DOI:** 10.1093/pnasnexus/pgac077

**Published:** 2022-06-09

**Authors:** Erik Brown, Igor Cavallini Johansen, Ana Paula Bortoleto, Yadu Pokhrel, Suyog Chaudhari, Anthony Cak, Samer Sulaeman, Laura Castro-Diaz, Maria Claudia Lopez, Adam Mayer, Judith Walgren, Norbert Müller, Emilio Moran

**Affiliations:** Department of Mechanical Engineering, Michigan State University, East Lansing, MI 48823, USA; Center for Global Change and Earth Observations, Michigan State University, East Lansing, MI 48823, USA; Núcleo de Estudos e Pesquisas Ambientais (NEPAM), Universidade Estadual de Campinas (UNICAMP), Cidade Universitária Zeferino Vaz - Barão Geraldo, Campinas - SP, 13083, Brazil; Department of Infrastructure and Environment. School of Civil Engineering, Architecture and Urban Design, University of Campinas, Rua Saturnino de Brito 224, PO Box 6143, Campinas, SP 13083-889, Brazil; Department of Civil and Environmental Engineering, Michigan State University, East Lansing, MI 48823, USA; Department of Civil and Environmental Engineering, Michigan State University, East Lansing, MI 48823, USA; Environmental Sciences Initiative, CUNY Advanced Science Research Center, New York, NY 10031, USA; Department of Mechanical Engineering, Michigan State University, East Lansing, MI 48823, USA; Department of Community Sustainability, Michigan State University, East Lansing, MI 48823, USA; Department of Community Sustainability, Michigan State University, East Lansing, MI 48823, USA; Center for Global Change and Earth Observations, Michigan State University, East Lansing, MI 48823, USA; School of Journalism, Michigan State University, East Lansing, MI 48823, USA; Department of Mechanical Engineering, Michigan State University, East Lansing, MI 48823, USA; Center for Global Change and Earth Observations, Michigan State University, East Lansing, MI 48823, USA

**Keywords:** renewable energy, off-grid, participatory design, convergence

## Abstract

While there have been efforts to supply off-grid energy in the Amazon, these attempts have focused on low upfront costs and deployment rates. These “get-energy-quick” methods have almost solely adopted diesel generators, ignoring the environmental and social risks associated with the known noise and pollution of combustion engines. Alternatively, it is recommended, herein, to supply off-grid needs with renewable, distributed microgrids comprised of photovoltaics (PV) and in-stream generators (ISG). Utilization of a hybrid combination of renewable generators can provide an energetically, environmentally, and financially feasible alternative to typical electrification methods, depending on available solar irradiation and riverine characteristics, that with community engagement allows for a participatory codesign process that takes into consideration people’s needs. A convergent solution development framework that includes designers—a team of social scientists, engineers, and communication specialists—and communities as well as the local industry is examined here, by which the future negative impacts at the human–machine–environment nexus can be minimized by iterative, continuous interaction between these key actors.

Significance Statement Current electrification methods are not sufficient to supply all people with power, or are environmentally, ecologically, and socially deficient. Millions of people are off-grid and lack affordable and clean energy access. Utilizing distributed, renewable hybrid off-grid electrification methods, specifically in-stream generators and photovoltaic panels, in the Amazon basin, is examined and shown to be energetically and financially feasible depending on locally available riverine characteristics. Further, the environmental and social appropriateness of these systems is considered, and conclusions about participatory processes for successful project implementation and long-term sustainability are made.

## Introduction

Globally, there will be approximately 660 million people without electricity access by 2030 (1). In the Amazon basin region of Brazil alone, 990,103 people were without power as of 2019 (2). Implementation strategies for increasing the electrification rate of off-grid communities in the Amazon region have been investigated in the literature, in addition to potential management structures and subsidy programs that can be utilized in order to support these systems (3–6). Often described as distributed mini-grids or microgrids, depending on their scale, these systems comprise different combinations of energy sources to allow for maximum reliability and availability: solar, hydraulic, wind (or eolic), combustion engines of various fuel types, and energy storage if needed. To increase the electrification rate in Brazil, with a goal of 100% electrification, the Brazilian government enacted a series of social programs, named PRODEEM and its successor Light for All (LPT from the Portuguese wording, Luz para Todos). While the PRODEEM program aimed to meet the electrification goals using renewables (i.e. solar panels), its implementation was not entirely successful, such as in the state of Amapá, where over 80% of the systems installed between 1998 and 2003 were rendered inoperative (7). After the PRODEEM program was converted to LPT, there was a shift in preferred generation methodology to primarily thermal generators. According to the National Electricity System Operator (ONS) of the Brazilian National Interconnected System (SIN from the Portuguese wording, Sistema Integrado Nacional), as of 2021, there are 212 isolated power systems in Brazil, 94.6% of which use diesel generators (8, 9). Additionally, in 2017, Siemens proposed installing 80 MW of diesel generators along the Amazon River to supply off-grid energy to 12 communities (10). A total of four of the 12 sites were reported to already have margin deficits (the difference between the generation level and the load) by 2019; the other sites did not have margins reported due to their planned interconnection to the SIN by 2023 (11). The choice to use diesel generators for off-grid energy is common for their relatively low upfront costs and quick deployability, however, they have several drawbacks: continuous fuel costs, greenhouse gas emissions from burning fossil fuels (12), environmental risk of spilled fuel during transport to sites, noise when used without proper insulation or when operated at large scales (13, 14), loss of fuel due to theft and leaking (14), and dependence on costly diesel that is sometimes provided by government subsidies (15). Given the environmental, ecological, and social concerns related to the use of fuel-powered generators such as diesel, an efficient, reliable, and more environmentally friendly mixture of energy sources is needed. A hybrid renewable in-stream generator (ISG)–photovoltaic (PV) system is considered in this work as a feasible solution that overcomes the drawbacks of fuel-powered generators while meeting the household needs. In tandem, social considerations are also included, to set the groundwork for successful implementation of real-world projects with the engagement and participation of local communities, industry, and designers (social scientists, engineers, communication specialists, and regional and federal governments).

## Hybrid Energy Sources

**Fig. 1.**
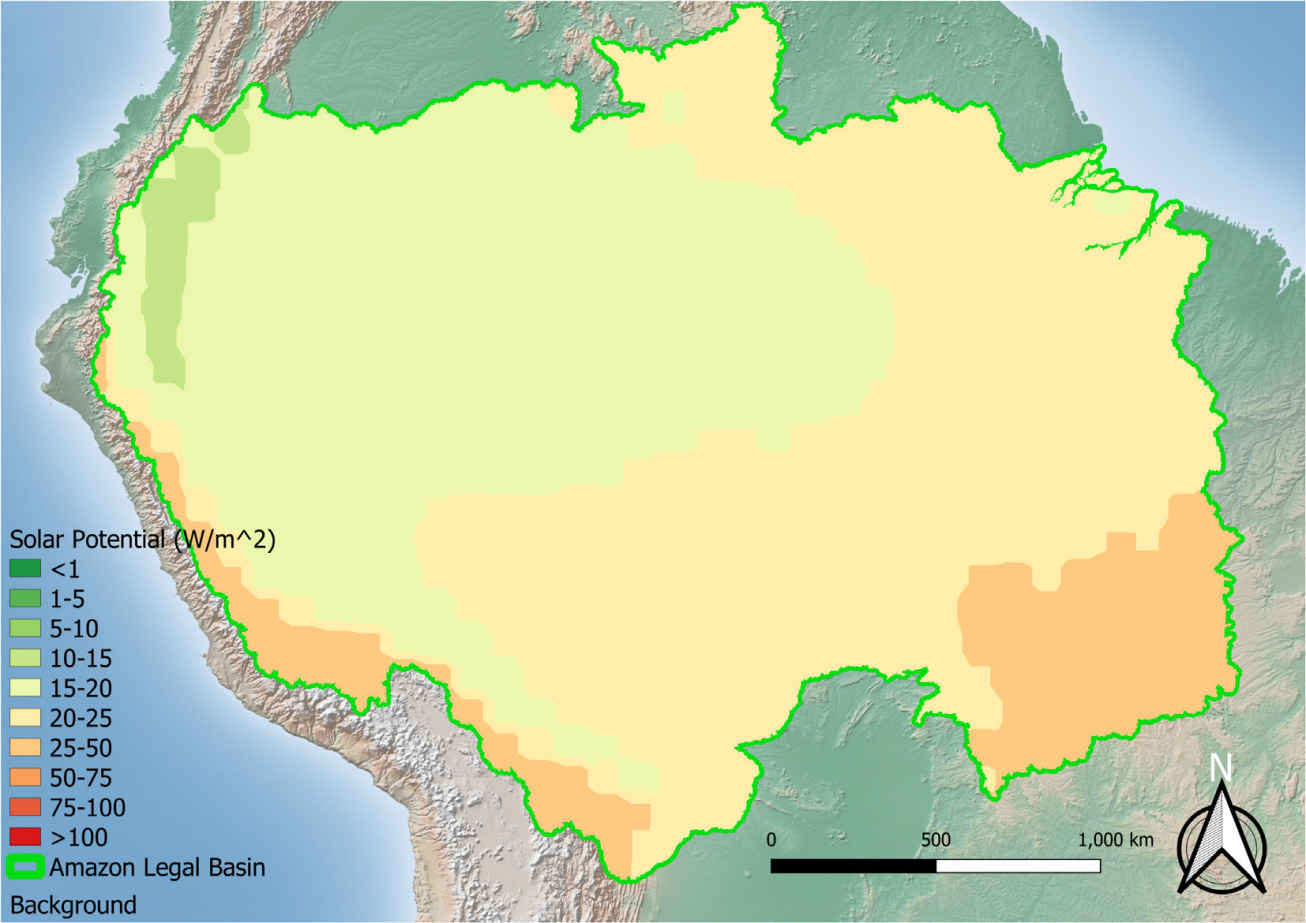
The direct normal solar irradiation potential in the Amazon, assuming an overall 12% conversion efficiency (data source: (18)).

**Fig. 2.**
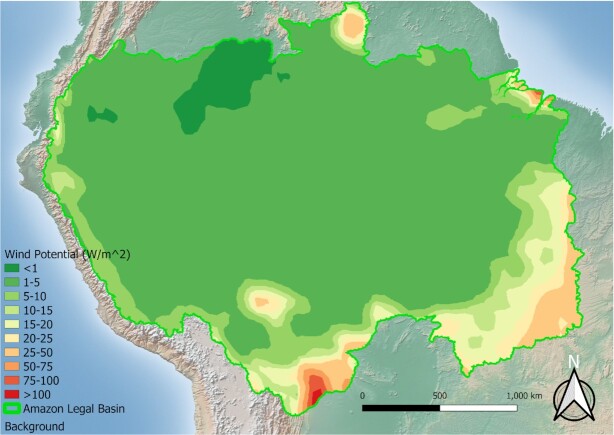
The eolic potential in the Amazon at a height of 50 m, assuming an overall 45% conversion efficiency (data source: (18)).

A hybrid generation system is used to decrease costs per watt, increase reliability, increase environmental friendliness (by decreasing proportion of a single resource being utilized), and increase the overall system capacity factor (the amount of time energy is available). The mixture of energy sources will vary by region and the location of the communities relative to any geographic-based resources (river velocity, open ‘sunny’ areas, agricultural or other natural residues, underground hot springs, and so on). In the Amazon basin the renewable energy potential is vast, and has scale-based decisions to determine the optimal mix. The Amazon’s hydraulic potential is among the highest of the global basins, in both utilized and estimated total potential (16), especially in the Madeira, Tapajós, and Xingu sub-basins (17). As shown in Figs 1 and 2, the PV potential is high relative to the wind potential at a height of 50 m for most of the Amazon basin. This relatively high PV potential has given rise to increased global attention and funding for PV, specifically floating PV systems to augment existing hydropower, for grid-tied applications and water conservation efforts (19, 20). A mixture of in-stream and solar energies is also important for off-grid systems in the heart of the Amazon basin, while for projects that may be closer to the Brazilian coasts, wind may be a more promising candidate. For hydropower production, it is recommended to utilize in-stream (or hydrokinetic) generators instead of the traditional dam-based generation (21). This recommendation is made because of the potential issues associated with large-scale hydropower generation (22), and because most communities in the Amazon region are located near rivers. Additionally, biomass combustion is a potential option; however, as to not incur environmental issues associated with large-scale bioproduction (deforestation for farmland or pasture, loss of biodiversity, and so on) (23–25), it should be limited to regions where the natural sources are present or used only at very localized scales (existing agricultural byproduct, riverine floating wood, and so on).

## Off-Grid Generation System

The question of who needs power as well as how much is needed is naturally accompanied by the question: why is power needed? There are two main answers to this question from a design perspective: the first short answer is that the household has little to no access to power and desires access for devices such as lights, refrigeration, cell phones, and television, or for community communal uses: lights for a school, electrical devices in a hospital, internet access, computers, and resources for fishing cooperatives. The second answer is expanding financial interests: a need for power to increase economy, manufacturing, distribution, and/or efficiency. Though both answers indicate power needs, the method to provide power and the amount needed can be very different. This difference is polarized in the Amazon due to a portion of the population living in highly geographically removed rural regions, possibly deep within the rainforest itself, and far away from any city. The distribution of electricity to these populations living far from the national grid becomes difficult and costly for the federal government, private entities and households themselves, both financially and environmentally. For communities close to the current electric grid, or those who will be close to future line extensions, it is recommended to provide them with a direct tie-in to the grid. However, for the people who are far from the current national grid, it makes more sense to utilize natural resources in the nearby region to produce power locally. Thus, an off-grid energy generation solution is necessary.

**Fig. 3.**
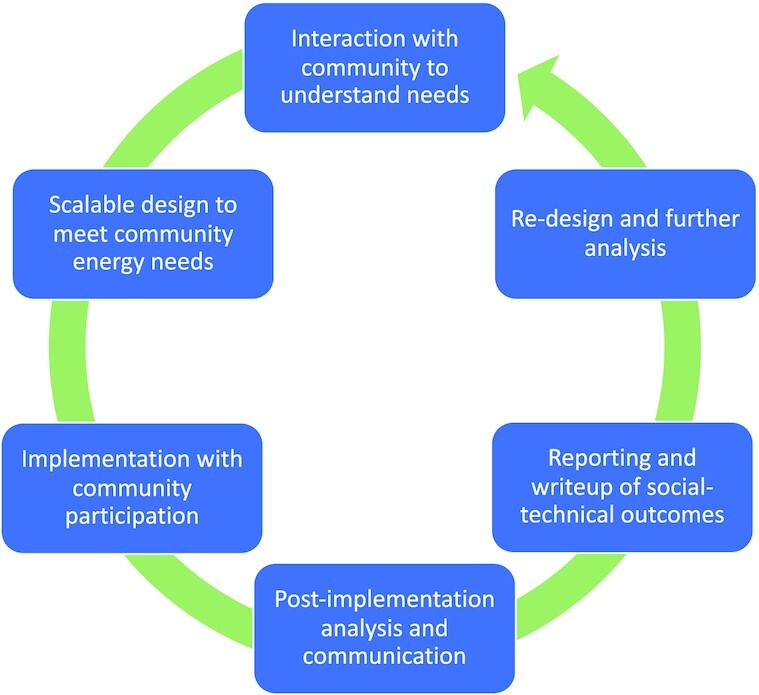
A simplified form of the iterative design and reporting process for participatory design.

To determine a generation system that best fits the needs of the people benefitting from the work, a participatory iterative process is required, shown in Fig. 3. The starting point in the cycle is a dialogue with the communities. From the initial conversations, the needs of the community are to be understood: the individual household electricity needs, the communal electricity needs, and the daily habits of energy usage if electricity has been available previously. Once the needs are known, and the community environmental, ecological, and social characteristics are identified, the details of options for a generation system can be developed. After the energy options are explored and the feasibility studies performed, another dialogue with the community should inform the members how much energy can be made available based on their local resources. With the system output estimated and communicated, the community will be able to plan how the energy will be allocated, and how to best set up the system in the community. The cycle will then continue until the designer, the community, and any other involved actors (such as the local industry that will provide the devices) are optimally satisfied with the system design.

### Load model development

**Fig. 4.**
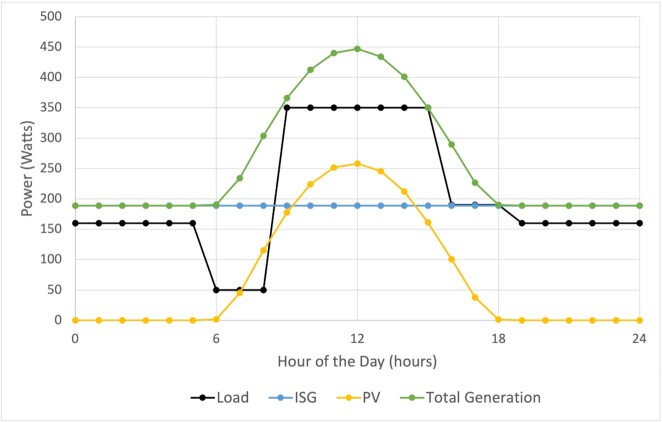
Scaled community load profile with suggested generation profiles. The black curve is the load, and the green curve is the total generation, which is the summation of the blue curve (the in-stream generation) and the yellow curve (the solar generation).

A load model is the cumulative electricity requirements at any given time, where the first stage of developing a load model is to select devices that are “common needs” for the communities of interest. In the process of load development for remote regions, two cases are considered: communities that have had no previous electric access (a previously unpowered community, UPC), and those that have had some level of electrification (a previously powered community, PC). The difference between the two cases is the likelihood of expectations for device usage; namely, communities that have had electric access may be accustomed to having more devices in use throughout the day. Conversely, communities that have had no electricity access may be more willing to initially accept a reduced load, which may be more beneficial if piloting a project is the initial goal. For the case of Amazonian off-grid communities, the following devices were chosen to constitute the load model: a refrigerator, lights, a fan, a television, and a small standalone freezer. A PC case would include one or more additional high consumption device such as an electric shower heater. With the devices known, the next step is to assume device instantaneous power requirements and daily usage habits for the devices. A load curve is created by adding the cumulative power draws of the devices at each hour of the day and then introducing load management concepts into the modeling process to reduce costs. An example of a management system in its simplest form is using outlet timers to regulate the usage of a device, especially those with large power draws, like the refrigerator, resulting in a load such as the one shown in Fig. 4. For the case of the refrigerator, this could mean only turning on the compressor during the day to keep it on just long enough to keep the contents (for example, water) cool until the next day, whereas more quickly perishable items, like fish or fruit, could be kept in a freezer that runs constantly. This method allows the peak load to occur only during the day such that the PV component of the hybrid mix can be effectively utilized without the need for batteries to meet the peak demand.

### Generation determination

Generation costs were calculated over the range of 1 to 2.8 m/s of river flow speed, with 2.8 m/s as the maximum for the ISG’s rated maximum power level (see Supplementary Material Section 1 for more information), assuming that there is cut-out control. The results are shown in Fig. 4, where the load models are shown with their optimally low equipment cost generation curves, as well as the calculated base equipment cost. Profiles with an optimum generation mixture consisting of both PV and ISG (as opposed to pure ISG or PV) often generate more energy than load demand, leading to lost power unless an isolated energy storage system is used or this excess energy is sold back to the grid. However, this energy can be repurposed without batteries, such as making ice by using a split refrigeration system.

### Financial analysis

**Fig. 5.**
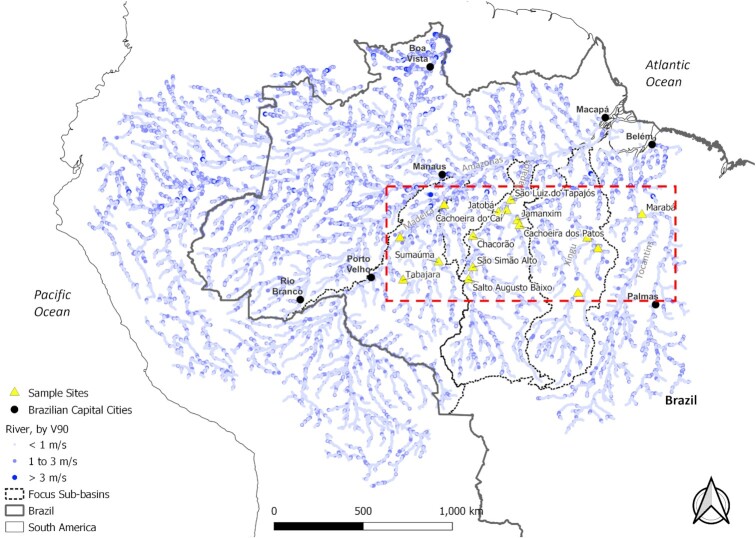
Locations of sample sites overlaid on top of river paths, with markers representing the river velocity available at that location, based on *V*_90_. The sample sites are chosen to explore the range of cost within the focus region (encompassed approximately by the dashed red line); the sites that correspond to potential planned dam sites are labeled.

The energetic systems considered as a basis for financial inputs are described in Supplementary Material Section 2. To calculate the costs of the proposed system, the placement of both the PV panels and the ISGs have to be specified. For the PV panels, it is assumed that they will be placed locally on the rooftops of households or common buildings. For the ISGs, the optimal scenario for reduced costs is a community located within approximately 50 m of the edge of a river with high flow velocity water, where the cabling provided could transmit power to shore. However, as it is possible that most communities are not proximate to river locations with high flow velocity, an alternative solution to keep costs low and power capacities closer to rated levels is for in-stream devices to be placed further away, with power transmitted by longer cables (likely buried shallowly in the ground to avoid deforestation). With longer cables, costs will increase in proportion to the generator system rating as well as the distance to the nearest high-velocity river location. To explore the possible range of the proposed solution costs in the Amazon, several sample sites shown in Fig. 5 were chosen based on currently planned dams as well as planned dams that were halted for environmental/social reasons (26–28). Additional sites were introduced to distribute the sample locations more evenly among the Northern regions of the three focus sub-basins (Madeira, Tapajós, and Xingu). The sample sites act as proxies for communities potentially present at these locations and serve as case studies for examining how a mixture of ISG and PV can be a viable alternative to large-scale dams, transmission line extensions, and diesel generators for providing local power.

**Fig. 6.**
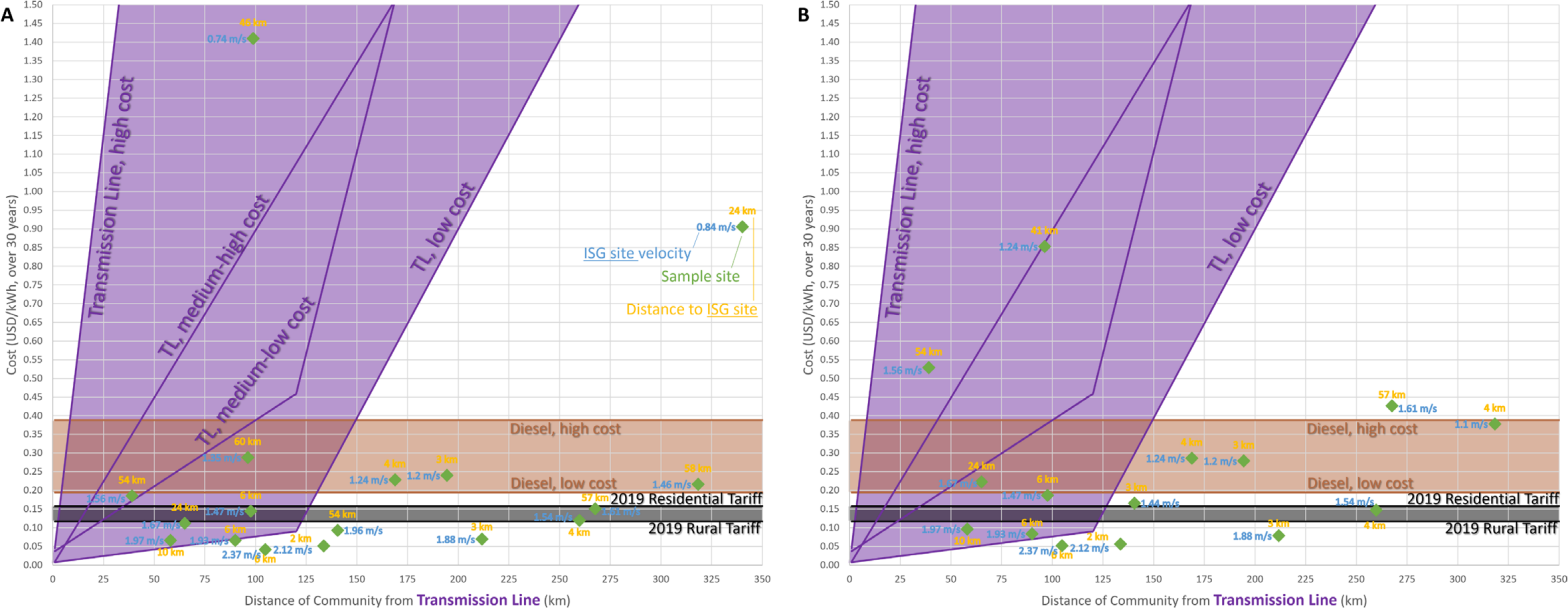
Comparison of proposed UPC solution (green diamonds) with several common energy generation and distribution methods with cable costs of: (A) 1,000 USD/km and (B) 6,000 USD/km. Costs associated with electric grid extension is shown by the purple range, a diesel generator is shown by the brown range and the 2019 Brazilian North region residential and rural tariffs are shown by the black range. The *x*-axis is the distance from the community to the nearest existing transmission line, in km, and the *y*-axis is the calculated cost in USD/kWh over 30 years.

**Fig. 7.**
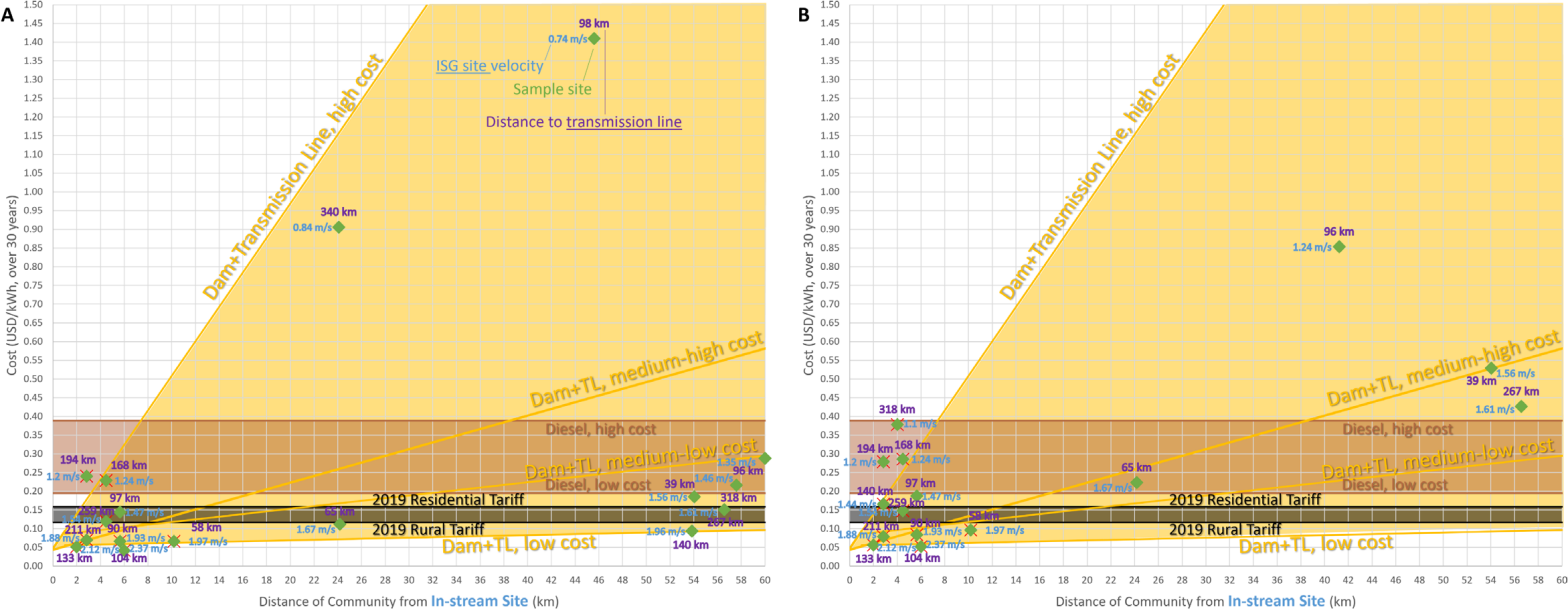
Comparison of proposed UPC solution (green diamonds) with several common energy generation and distribution methods with cable costs of: (A) 1,000 USD/km and (B) 6,000 USD/km. Dam construction with distribution lines is shown by the gold range, with no underreporting factor included; the other ranges are the same as Fig. 6. The *x*-axis is the distance from the community to the optimal in-stream generation site, in km, and the *y*-axis is the calculated cost in USD/kWh over 30 years.

The costs of the proposed solution at these sample sites are compared to the cost of common electrification methods, detailed in Supplementary Materials Section 2. Figs 6 and 7 show the results of the microgrid sizing calculation for a previously unpowered community (UPC) by comparing the calculated proposed solution costs against the calculated costs for an electrical grid extension or new dam construction with distribution lines, respectively, where a red x symbolizes a possible community flooding/displacement, depending on the size of the reservoir built for the dam.

## Environmental, Ecological, and Social Considerations

### Environmental and ecological considerations

ISGs have been noted as being environmentally safe and ecologically safe (29, 30). ISGs are a more environmentally and ecologically friendly option than dams, as no reservoir or barrier is required for their operation, allowing most of the riverine flow to naturally bypass around the device. With no reservoir or barrier required, fisheries are not blocked from their natural migration patterns; sediments and debris are not trapped and are free to flow downriver, thereby decreasing greenhouse gas emissions compared to other tropical dams; toxic substances do not build up as can happen in the hypolimnion of reservoirs; and, evaporation rates are not increased over natural riverine levels (31–33).

In addition to the effect of the ISG on the surrounding environment, the inverse should also be considered. For example, flow velocity will be influenced by any natural or anthropogenic changes in the basin, such as droughts or floods, or the building of new dams. While it is unclear a priori how such changes in river flow will affect ISG operations, the effect could be in one of three major cases: a positive increase in unit power, no change, or a negative change. Namely, depending on the location and extent of the future riverine disturbance, the river velocity could increase, stay nearly constant, or decrease over a given period, with generator output following accordingly. Another potential effect of the natural environment on the generator unit is biofouling. Tropical basins such as the Amazon are rich with organic matter, which will accumulate on the device, limiting its output. ISG provide a thrust force against the flow direction, and thus naturally allow most of the natural riverine flow to bypass the device, along with fish and larger debris. Additional generator design considerations can be used to mitigate small and large debris build-up, including diverging trash racks and backwards swept frontal features that ensure as much organic material as possible continues to flow downstream.

Another major issue to consider is energy storage, most importantly, the disposal of energy storage devices. Environmentally safe batteries, such as saltwater-based technology, can be expensive upfront to purchase and are not widely available, but offer the benefit of being nonflammable and nontoxic (34), while cheaper, standard lead–acid batteries can be environmentally damaging at the end of their life, if they are not properly recovered. Common disposal methods are for them to be buried in dirt or water if there are no actions toward physically removing or replacing them, as is the case for Amazonian communities. Similarly, solar panels need an end-of-service plan for the specific community in question, to avoid being disposed of improperly: recycling will require that the local industry maintain active participation during the life of the project to ensure its full success.

### Social considerations

There are several social elements that are crucial for successful implementation and sustainability of a new power system in a community, including but are not limited to:

- Community leadership
- Scalability of participatory design process and maintenance
- Adaptability of “off-the-shelf” energy systems to community needs
- Social commitment toward the project
- Population dynamics (e.g. new households)
- Energy use behavior changes after system installation
- Producing a realizable plan for system maintenance and end-of-life action.

For example, changes in energy use behavior in a community occurs when the installed system allows for flexible 24-hour use. After years of limited access with diesel generators (e.g. 4 hours each day), a rebound effect dictates that consumers will tend to consume more (e.g. doubling TV watching from 4 to 8 hours per day, and so on) (35–38), possibly up to the point of generation not being able to meet load demands. Compounding this direct effect is the indirect effect of more devices being purchased that will consume more electricity (35), leading to further stress on the electrical system. Corresponding increased industrial production of goods will also stress not only the electrical system of a community but its surrounding environment. To target this issue, researchers and community members must work together to establish energy conservation habits according to social–ecological and technological system characteristics such as the seasonality of natural resources (especially water speed and solar incidence variability during the year) and community livelihoods. Riverine community energy needs are also not universal; some communities rely on fish production while others are more devoted to agriculture or economic activities requiring differing energy load levels. This diversity reflects a need for unique and localized strategies involving active participation between designers, community members, and local industry in order to implement a successful and sustainable off-grid power system. It should be noted that providing electricity for those in need is not only a matter of solving the access issue in isolated communities; it is also about providing quality of life and empowering social development for all. This occurs since energy availability improves population health, food security, and access to education (39–41).

**Fig. 8.**
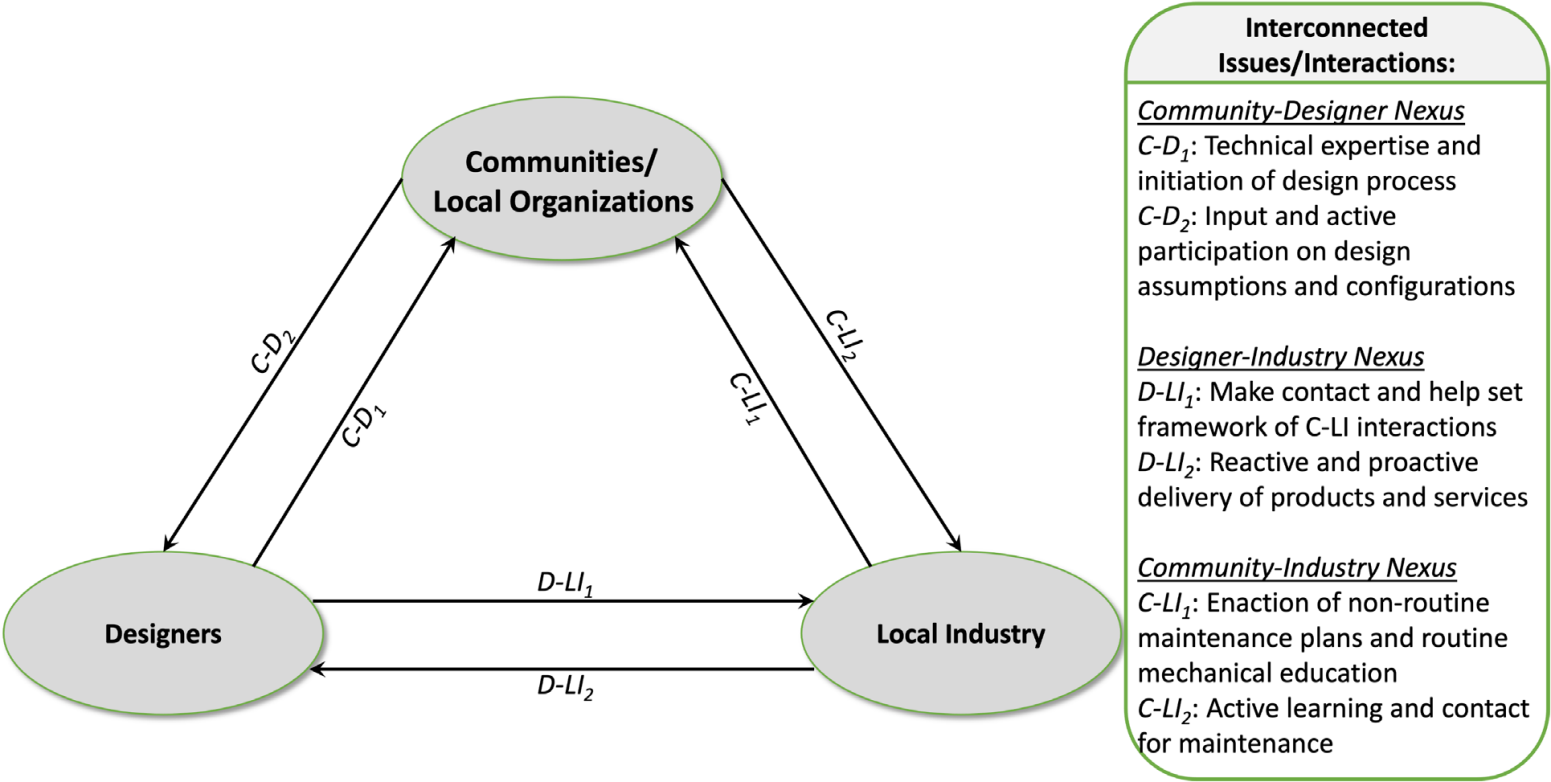
Interaction network of stakeholders in participatory microgrid projects.

Such issues highlight the importance of one central concept: a need to engage and cocreate with communities, local organizations, and local industry. Previous research has shown how the lack of engagement and participation of local communities in energy projects have generated negative impacts, which reflects the need of more equitable and just energy projects (42). While these considerations are not exhaustive of the full details that need to be considered, they constitute a critical subset of issues that spans the design and implementation process. The issues are examined here in brief, and it is hoped that many can be addressed as the literature becomes richer in successful, convergent social–ecological–engineering deployments. These issues cannot likely be solved with punctual interactions, but require an inclusive and iterative engagement and long-term commitment to enable successful project implementation; see Fig. 8 for the interaction nexus.

The actors may vary for each specific community, but one example is: the design team is made up of the researchers intending on implementing the project, the community/local organization are all of the members of the community that want to participate, but could include at a minimum a leader and a maintenance contact, and finally, the local industry is any local partner that is involved in providing any physical product or service through the design team or directly to the community. This example likely models the first novel interaction with a community, where a system design is being investigated that is sought to be generalized to other communities. For applying the system and solution to other (eventually, all) communities, a different set of actors is required: the design team is taken over by government workers, likely from the local electricity agency or the state itself, and the local industry may be enhanced by supply chains containing larger-scale providers, such as government-bid energy equipment providers. This interaction nexus should, thus be considered by both researchers seeking to determine and pilot new solutions, as well as the state and federal government entities that need to provide for the public good, for which the success of a project should be measured in terms of the social and economic benefits achieved from electricity access (43). Electricity is elementary to social development in the Amazon and it is needed to assure the rights of education, health, food, work, housing, leisure, and security, guaranteed by the Brazilian Constitution of 1988 (44).

## Discussion and Conclusions

The cost outcomes of a proposed hybrid renewable ISG–PV system are shown in Fig. 6, which examines costs for electrifying a previously UPC with this system as compared to an extension of the current electric grid or a use of a diesel generator. Also shown are two electricity tariffs for rural and residential areas of the Brazilian North region (which do not vary significantly between 2019 and 2021). A range of outcomes were calculated for two cabling scenarios: low cable costs (1,000 USD/km) and high cable costs (6,000 USD/km). Variation in calculated cost outcomes was largely dependent on local river velocities and community distance to rivers and/or the current electrical grid.

For most off-grid rural locations (approximately 58% of sample sites) in the low cable cost scenario, hybrid energy costs were lower than rural tariffs; these locations, which were associated with river velocities above 1.54 m/s, may benefit most from the proposed hybrid energy solution. Increasing this number of locations to include those whose costs were found to be in the range of the two tariffs (83% of the sample sites) also showed that the proposed hybrid system has lower costs that diesel generation or grid extension. Low river velocities inhibited financial benefits of the hybrid system for two locations (0.74 and 0.84 m/s), with hybrid system costs greater than diesel generation. Connection to the electrical grid was potentially beneficial for only three sample locations, and dependent on whether low extension costs could be maintained with no major additional substation or electrical infrastructure needed to support the increased load; these locations were found to be approximately 90 to 100 km from existing transmission lines.

For the remaining off-grid residential areas, hybrid energy generation costs were in the range of the residential tariff and the high cost threshold for diesel generation; such examples show that a hybrid system can be competitive with diesel generation for supplying power to these types of off-grid communities. Consequently communities that still need power could benefit from this proposed hybrid solution and not depend on fossil fuels or government fuel credits. Increasing the cable cost from 1,000 to 6,000 USD/km yields the expected result of increased costs associated for in-stream generation; without the presence of a battery, the total project cost of energy also increases. For most locations with costs comparable to tariffs or diesel generation, though, an increased cable cost did not affect the overall pattern or outcome, in that hybrid systems were still competitive with diesel generation or grid extension. Only two locations with already high-cost outcomes (greater than the high-cost threshold for diesel generation) remained unfeasibly high (so high that they were greater than what could be plotted on the figure’s axis).

Across these different scenarios, it is clear that hybrid, renewable grids can serve as successful alternatives to common electrification methods. While they have been shown to be more environmentally and socially friendly than other methods, especially for providing opportunities for community engagement and the system ownership and management (i.e. maintenance), importantly they also have been shown to be financially feasible, depending on design constraints and site-specific needs of individual communities. In order to increase the likelihood of success of an off-grid project, local communities and local industry (manufacturers, energy agencies, local governments, and so on) must be included from the very beginning and throughout the design and implementation process, in what is now known as a convergence approach to problem-solving. Understanding and documenting the needs, behaviors, and interactions of the community, industry partners, and the designers/researchers, and any changes in those social variables (as well as any technical or political variables) can help determine the drivers for the success of the project, or its failure—mechanisms that can help develop a base for real-world participatory design or codesign processes. Future work to develop these convergence concepts alongside real project implementation is crucial in understanding and advancing technologies and strategies at the human–machine–environment nexus to bring a better quality of life to all people, while protecting the planet for future generations.

## Funding

This work is supported by the National Science Foundation grant number 2020790 (GCR: Convergence for Innovative Energy Solutions) and the NSF grant number 1639115 (INFEWS: Rethinking Dams: Innovations at the Nexus of Food, Energy, and Water Systems).

## Authors' Contributions

E.B.: paper conceptualization and drafting and data processing; I.C.J.: editing and conceptual enhancement; A.P.B.: editing; Y.P.: editing and project comanagement; S.C.: editing; A.C.: editing; S.S.: editing; L.C.-D.: editing; M.C.L.: editing; A.M.: editing; J.W.: editing; N.M.: project comanagement; and E.M.: editing and project management.

## Supplementary Material

pgac077_Supplemental_Files

## Data Availability

The datasets (Microsoft Excel Workbooks and VBA scripts) generated during and/or analyzed during the current study are available in the Dryad repository accessible at: https://doi.org/10.5061/dryad.rn8pk0pc9.
