# Supplementary material for: Feasibility of hybrid in-stream generator–photovoltaic systems for Amazonian off-grid communities
Source: PNAS Nexus. 2022 Jun 9;1(3):pgac077. doi: 10.1093/pnasnexus/pgac077 (PMC9896937; doi:10.1093/pnasnexus/pgac077)
Supplement: pgac077_Supplemental_Files [file pgac077_supplemental_files.zip › PNASNEXUS-PNASNEXUS-2022-00170-s01.pdf]

Supplementary Information for

Feasibility of Hybrid ISG-PV Systems for Amazonian Off-grid Communities

Erik Brown, Igor Cavallini Johansen, Ana Paula Bortoleto, Yadu Pokhrel, Suyog Chaudhari, Anthony Cak, Samer Sulaeman, Laura Castro-Diaz, Maria Claudia Lopez, Adam Mayer, Judith Walgren, Norbert Müller, Emilio Moran

Erik Brown.
E-mail: brown722@msu.edu

This PDF file includes:

Supplementary text
SI References

Supporting Information Text

All data and computational processes represented here and in the main text can be accessed via (1).

1. Generation Determination

After the load model is developed, the generation required to meet that load can be determined. Due to the energy potentials shown for the Amazonian region, it is recommended to utilize in-stream generators (ISG, hydraulic) and solar (PV, photovoltaic) energy sources. The number of in-stream generators depends on the maximum load during 'non-sunny' hours as well as constraints from the river: the average depth and transverse flow velocity profiles, the intermittency of availability of the flow velocity, the depth and width of the river, and the distance from the river to the community. The ISGs are to be placed in the highest velocity river stretches nearest to the community, and the power shared and distributed to the households and/or common buildings. The number of PV panels depends on the difference between the constant hydro-met load and the peak load, the time of day of the maximum load difference, and the specific coordinate location of the community. The PV panels are suggested to be placed on the roofs of the individual households or on top of central community buildings, both of which will provide the communities with the most convenient solution and provide the strongest feeling of 'self-ownership' for the solution; the individual people can feel that they were considered individually and directly.

To evaluate the detailed financial analysis, a Microsoft Excel VBA-based script was developed to: be able to test a wide range of conditions, automatically extract data from an internet source from Renewables.ninja (2), logically locate and tabulate river conditions from a Leaf-Hydro-Flood (LHF) hydraulic model (3), facilitate rigid solver input-output, and be able to more easily and consistently adapt to the solution output signals. User input is provided on an Excel sheet, in the form of the coordinates, household count, and if the community has had electric access previously (previously "powered" or "unpowered"). The coordinates are used to look up the hourly solar data over one year using another excel VBA script provided by Renewables.ninja (2) along with the size of the PV plant to design (1 kW), the desired solar model (MERRA-2), the tilt and azimuth of the planned panels (0 degrees: the conservative case that the panels are mounted flat), and the additional system loss (10%). The year of hourly data is averaged for each hour of the day, which is then scaled by the required hourly PV rated power at the current solver iteration. The PV rated power is used as a scaling factor over the 1 kW script input plant size because the solar data estimates the relative efficiency of the panel, utilizing a parameterized energy performance model presented by (4). The solar script uses the MERRA-2 global reanalysis database to extract the location-specific 2-meter displacement height air temperature (T2M, zero wind log-profile velocity) and irradiance to estimate the module temperature via an empirical relationship to calculate the module heating and relative efficiency (2). The relative power performance equation presented by (4) is then solved, reproduced here as Equations 1-4, using the free-standing module temperature rise coefficient, in Equation 2.

$$T_{mod} = T_{amb} + c_T * G \quad [1]$$

$$c_T = 0.035^oCW^{-1}m^2 \quad [2]$$

$$\eta_{rel} = 1 + k_1 \ln(G^{'}) + k_2\left[\ln(G^{'})\right]^2 + T^{'}\left[k_3 + k_4 \ln(G^{'}) + k_5\left[\ln(G^{'})\right]^2\right] + k_6 T^{'2} \quad [3]$$

$$P = P_{STC} * \frac{G}{G_{STC}} * \eta_{rel} \quad [4]$$

Where $G^{'} = \frac{G}{G_{STC}}$, $T^{'} = T_{mod} - T_{mod_{STC}}$, $G_{STC} = 1000W/m^2$, $T_{mod_{STC}} = 25^oC$, G is the in-place irradiance, and T_{amb} is T2M and $k_1 - k_6$ are reported in literature from experimental data (4).

Once the PV data is imported, the approximately 30-year average river flow rate and flow velocity if found for the closest river location to the community coordinate (found by using automated logic to search for nearest reasonably-high flow rate, $\mathcal{O}(100 - 1000)$). The river flow and cross-sectional area data comes from a LHF hydraulic model, carried out at a 2 km resolution for the entirety of the Amazon River basin (3). Once the nearest "large" river has been found, then the highest river velocity in the surrounding tabular region (found by using similar logic to the previous step to find tabular direction of river) is found, at which point the solver setup and iteration can proceed. The generator power is calculated via use of a curve fit from data available from SmartHydroPower (5), utilizing the river velocity found previously, or equal to the rated 5000 Watts at the full-rated 2.8 m/s. Equation 5 shows the utilized power curve fit, where P_{ISG} is the generator-included power of the in-stream unit, and C_∞ is the free-stream velocity found for the given river location. The in-stream unit is assumed to be able to operate at the calculated level 24/7, with the one caveat: because the Q_{90} value is used, for approximately one month of an average year there may be reduced flow velocities and thus reduced power output, where for the rest of the eleven months of the year, the Q_{90} value will be available or even exceeded.

$$P_{ISG} = \begin{cases} 196.43 * {C_\infty}^{3.1336}, & \text{if } C_\infty < 2.8 \ m/s \\ 5,000, & \text{if } C_\infty = 2.8 \ m/s \end{cases} \quad [5]$$

A nonlinear Generalized Reduced Gradient (nGRG) algorithm was utilized within a VBA environment, using Microsoft Excel's solver function. This allows the evaluation of generation to meet the load while optimizing for cost. The nGRG method is a "...nonlinear extension of the simplex method for linear programming" (6). According to (6) and (7), this algorithm seeks to solve a series of simplified, or reduced equations.

2. Financial Inputs

The financial analysis begins with pricing components. For the off-grid solar system, the following items were chosen as a financial reference setup:

- Peimer SG33OP panels (0.504 USD/Watt)
- Solarland SLB0103 universal tilt bracket (0.172 USD/Watt)
- Schneider Conext SW4024 inverter (0.440 USD/Watt)
- Assumed approximately 0.1 USD/Watt for cabling and couplings

The Peimar panels are rated at 330 Watts, and have 72 cells. The panels output at a maximum of 36.4 Volts DC (VDC), but likely at normal operating conditions (NOCT) will output closer to 30 VDC, which is within the 20-34 VDC input required for the Schneider inverter, without the assistance of a voltage transformer. The Schneider inverter allows for 120 or 240 V output, which can fit the needs of most existing off-grid devices. These items yielded a PV system cost of 1.04 USD/Watt. At the time of developing this solution, little data is available on the price of in-stream technology. In industry, only one ready-from-the-shelf price data point was found: SmartHydro, based in Germany, offers a complete off-grid ISG package for 12,490 Euro or 14,580 Euro, depending on the available flow velocity and desired mooring configuration (5). This price can be equated to, at maximum generator output, 2.69-3.44 USD/Watt. The next input is the cable cost associated with the ISG deployment site not necessarily being proximal to the community of interest. The SmartHydroPower unit comes with 50 meters of cabling, which may be sufficient if the generator is not far from shore right near to the community. Requiring the generator to be right next to the community is convenient, but not necessarily always the most energetically or financially feasible option. Thus, a maximum distance of 60 km is considered between the community and the ISG deployment site (approximately half of a typical medium voltage line maximum length), and a 10 American Wire Gauge (AWG) off-the-shelf spool would be used as a basis for costs (ranging from 0.82 to 1.18 USD/meter, or approximately 1 USD/m). The cable cost range examined here is 1,000-6,000 USD/km, representing using only a 10AWG cable bundle (or more likely, multiple cables) up to a low-cost distribution network (transformers, external conductors, and other electrical components). It is noted that the PV system is not under consideration when examining the cabling due to the proposed placement of the PV panels being on the roofs of the community, thus, will always be a short distance away from where the power is used.

3. Financial Analysis and Comparison

Combining the ISG and PV systems costs, and scaling by the unit load, it is calculated that the cost of the off-grid solution is between 1.34 and 3.01 USD/Watt. The cost for all reported dams in the Amazon Basin is approximately 3.67 USD/Watt, and even higher for the Brazilian Amazon at 5.50 USD/Watt (8). The proposed solution thus can be economically viable and advantageous alternative to dams; a more detailed analysis is presented to determine the spatial extent and span of this statement.

To have a basis for comparison with the calculated cost of the proposed solution, several possible common Brazilian energy generation or distribution methods were estimated: an extension of the current national electric grid, the building of a new dam with distribution lines, using a diesel generator, and also the 2019 Brazilian North region tariffs (9). Though the tariffs represent the average charge for electricity while interconnected to the national grid, it is recognized that this rate may not be accurate for communities that are far from the grid without the aid of extra government incentive programs, due to the large costs incurred to extend the grid over a long distance. Regardless, the tariffs act as a "golden standard" guide for the cost effectiveness of a project, in that if a proposed system could meet or be lower cost than the tariffs, it could be a strong incentive for further examination by the government decision-makers (in Brazil: the electric agency ANEEL, the environmental agency EPE, the electric grid operator ONS, etc.) for utilization even beyond off-grid communities.

The first mode of energy provision is an extension of the national grid via a new transmission/distribution line network. To calculate the grid extension costs, two major perspectives to calculate the line costs were developed: 1) use Brazilian government reported data as a situational absolute; if the current method to supply any region with power is to build 230 kV line up to the point of distribution, then that is likely how it will be done for grid stability and reliability, and so the perspective is an accurate basis or 2) use a range of electrical component (high voltage (HV), medium voltage (MV), and low voltage (LV) lines, transformers, etc.) pricing from the literature to model a transmission system. For both scenarios a wide possible range of line costs were found, from approximately 6,000 USD/km, attempting to develop a cheaper low-to-medium voltage distribution/transmission line (10), up to 80,000 - 410,000 USD/km, calculated from existing and planned HV transmission lines in the Brazilian Amazon region ((11), (12), (13)). It is unclear at this time which value would be most representative of extending a transmission line deeper into the heart of the Amazon, where existing roadways and construction right-of-ways have not yet been established, the terrain may be highly variable, and native lands and protected areas may be present that prevent "shortest route" building, etc., thus a range of possible costs is considered.

The Brazilian government reported line costs were used on their own as total cost per kilometer, due to no additional information being provided about whether or not these are total project costs, or some subset of the total. Thus, the Brazilian cost estimates will be dubbed "total line" costs, as the line costs are assumed to capture all transmission costs, as opposed to just a "line" cost, which here is meant to refer to the cost of the cable alone. Additionally, substation costs were also not

included, assuming that the distances and loads will not be significant enough for substations to be required. From Brazilian data, it is calculated that an HV line could extend anywhere from 4 to 168 km (on average 77 km) ((11), (12), (13)) between substations. As long as the HV line does not extend far outside of the approximate range of 77-160 km, the assumption of not pricing a substation should hold. Equation 6 shows the calculation of absolute grid extension costs in USD:

$$C_{ONS_{tot}} = CC * L \qquad [6]$$

where $C_{ONS_{tot}}$ is the total grid extension cost based on data from the Brazilian government entity ONS, CC is the cable cost per kilometer calculated from the data, and L is the length of cable extension.

The literature-based component costs were used differently: instead of assuming that the cable cost is the “total line” cost, the cable costs are only one component of the whole system, which is built up from the following:

- HV, MV, and LV cable costs
- transformers
- household conversion equipment and connection costs

The LV line costs are calculated from an assumed inter-house distance of 25 meters (14) with a cable cost between 10,611 USD/km and 12,000 USD/km ((15),(14)), and the MV line is assumed to carry the power over a “typical” maximum distance of 120 kilometers to the community (16) at a cost between 6,000 USD/km and 30,580 USD/km ((10), (17)) at which point the HV line makes up the remaining distance, with a cost ranging from 90,000 USD/km to 192,000 USD/km, depending on operating voltage (16). The transformers are assumed to cost between 39 and 1,000 USD per rated kW ((15), (14)), the household equipment is assumed to cost between 263 and 367 USD per household ((15), (14)), and the household connection cost is 149 USD per household (15). The operation and maintenance costs are assumed to be between 2-3% for the transformers and cables, respectively ((15), (14)). The transformers are assumed to have 18% losses and a 10 year lifespan (15). The total cable length can be calculated by Equation 7, and the total grid extension costs were calculated with Equation 8.

$$L_{total} = L_{LV} + L_{MV} + L_{HV} \qquad [7]$$

Where L_{-} is the length of cable for a particular voltage category, or total length.

$$C_{COMP_{tot}} = \begin{cases} (1+OM_{MV}) * CC_{MV} * L_{MV} + ... \\ (1+OM_{LV}) * CC_{LV} * HH * d_{ihs} + ... \\ (1+OM_{tr}) * (1+LF_{tr}) * (T/t_{tr}) * (C_{tr} * P_{peak} * HH), \\ \text{if } L_{MV} \leq 120\, km \\ \\ CC_{HV} * L_{HV} + (1+OM_{MV}) * CC_{MV} * 120 + ... \\ (1+OM_{LV}) * CC_{LV} * HH * d_{ihs} + ... \\ (1+OM_{tr}) * (1+LF_{tr}) * (T/t_{tr}) * (C_{tr} * P_{peak} * HH), \\ \text{otherwise} \end{cases} \qquad [8]$$

Where OM_{-} is the operation and maintenance cost of the particular equipment in percent of cable cost, CC_{-} is the cable cost of a given voltage category found in literature in USD/km, HH is the number of households examined, d_{ihs} is the inter-household distance, LF_{tr}, t_{tr}, C_{tr} are the loss factor, lifetime, and unit cost of the transformers in percent, years, and USD/kW, respectively, and P_{peak} is the peak rated load power level.

The next mode of energy provision to compare the proposed solution to is the construction of a new full-scale dam with transmission lines built to bring the power to the community. Equation 9 shows the total estimated cost calculation of dam construction and power transmission.

$$C_{dam\,tot} = C_{dam} * UR * P_{peak} * HH + OM_{dam} * P_{MWh} + CC_{BR} * L \qquad [9]$$

Where C_{dam} is the average calculated cost of dam construction (5.5 USD/W) in the Amazon Basin and Brazil ((18), (8)), UR is the average global dam construction cost typical underreporting ratio (values of either 1 USD/USD or 1.96 USD/USD (19) were considered here), P_{peak} and P_{MWh} are the peak power and energy usage over a year in W and MWh, respectively, OM_{dam} is the operation and maintenance for a dam (2.31 or 5.8 USD/MWh) (20), CC_{BR} is the cable cost for transmission, assuming that the current Brazilian method for power transmission applies, and HV cables will be used for most of the distance, and L is the distance of cable. As was mentioned for the grid extension calculation, the Brazilian transmission line costs are used at the total line cost, assuming that all costs are included (right-of-way, projected operation and maintenance, etc.), due to a lack of information on project cost components. Both the grid extension and the dam construction use the Brazilian line costs, because unlike with a decentralized microgrid, the reliability of the national systems (whether it is a new dam or the grid itself) is under closer scrutiny by any interests-at-large, and so it is assumed here that the traditional HV line will be installed to reduce risk of outages if the load spikes become too large. Similar to the grid extension, a transmission network was also calculated from components in literature, which yields a cost calculated by Equation 10.

$$C_{dam_{tot}} = \begin{cases} C_{dam} * UR * P_{peak} * HH + OM_{dam} * P_{MWh} + ... \\ (1 + OM_{MV}) * CC_{MV} * L_{MV} + ... \\ (1 + OM_{LV}) * CC_{LV} * HH * d_{ihs} + ... \\ (1 + OM_{tr}) * (1 + LF_{tr}) * (T/t_{tr}) * (C_{tr} * P_{peak} * HH), \\ \text{if } \; L_{MV} \leq 120\,km \\ \\ C_{dam} * UR * P_{peak} * HH + OM_{dam} * P_{MWh} + ... \\ CC_{HV} * L_{HV} + (1 + OM_{MV}) * CC_{MV} * 120 + ... \\ (1 + OM_{LV}) * CC_{LV} * HH * d_{ihs} + ... \\ (1 + OM_{tr}) * (1 + LF_{tr}) * (T/t_{tr}) * (C_{tr} * P_{peak} * HH), \\ \text{otherwise} \end{cases} \quad [10]$$

The rating of a "full-scale" conventional dam that could be installed in the same river location as the in-stream site is calculated by Equations Eq. (11) and Eq. (12) were utilized along with the hydraulic data used to calculate the river velocity. The total head is calculated from the sum of the static head (water height, h_{-} and elevation, z_{-}) across the site, and the kinetic head at the site. This is converted into an equivalent total pressure and multiplied by the flow rate (Q_{90}) at the site to obtain the maximum theoretical extractable riverine power.

$$H_{total} = (h_2 + z_2) - (h_1 + z_1) + \frac{{C_\infty}^2}{2g} \quad [11]$$

$$P_{dam} = \rho * g * H_{total} * Q_{90} \quad [12]$$

Where ρ is the density of liquid water, 998.2 kg/m^3, and g is gravitational acceleration, 9.81 m/s^2. Only a portion of the total power calculated will be diverted towards the community, whereas most of the power is assumed to be directed to the existing electric grid. It is assumed that the community would then be charged only for the power utilized, equivalent to the cumulative community loads, $P_{community} = \frac{HH * P_{peak}}{P_{dam}} P_{dam}$.

One major difference between the grid extension and the dam construction is the risk of flooding and associated community displacement once the reservoir is filled. To estimate the reach of the reservoir, the average reservoir radius was calculated with respect to the nameplate capacity of each dam using Equation 13:

$$R_{res} \approx \sqrt{1.58 * P_{nameplate}/\pi} \quad [13]$$

Where $P_{nameplate}$ is the calculated theoretical capacity of the dam in MW, and 1.58 is the calculated average conversion for Brazilian Amazon dams in km^2/MW. With the reservoir area determined, a constant radius circular shape is assumed, neglecting the local topography, allowing for simple calculation of the reach of flooding. If the community is within the radius of the calculated reservoir, then it is concluded that it is possible that there is a risk of flooding for that community, and installing a full-scale dam at the in-stream site could lead to displacement of that community.

The last mode of energy provision examined is a diesel generator. To calculate the cost of energy of a diesel generator, typical usage from an Amazonian community in the state of Amapa was calculated. From (21), using the power rating (90 kW) and yearly allotted fuel consumption (27,600 liters), an estimated average load of 75% was calculated using power-consumptions curves/tables for similarly sized diesel generators (80-100 kW) at a fuel rate of ∼4.99 gal/hr. From this generator load, the number of households that the device could equivalently support was calculated, based on a UPC peak load of 350 W and 1,490 W for PC, yielding 192 and 45 households, respectively. There are two usage cases considered for a diesel generator: meeting the same load pattern as the proposed solution, and meeting an all-day constant, all-devices load (500 W for 135 households and 1,540 W for 43 households, for UPC and PC, respectively). The latter is considered as the "best-case scenario" for having constant access to all devices all day; however, the fuel costs would likely be too expensive for many households, and as was mentioned in (21), would not be funded in the fuel allowance (likely to be granted enough fuel for approximately 4 hours per day of usage, not 24), and so this case is considered as a theoretical lower limit for the given examined conditions. The diesel generator was quoted to cost 44,151.52 USD (21). To calculate the fuel costs, the Brazilian 2019 average diesel cost (22) was converted into USD, which was found to be 2.60 USD/gal. The cost of diesel generator is shown in Equation 14.

$$C_{dieseltot} = C_{generator} + CR_{fuel} * 2.60 * 8760 * T \quad [14]$$

Where CR_{fuel} is the consumption rate of diesel fuel by the generator.

The costs are calculated in USD/kWh, which was done for the UPC case by dividing the total costs by the expected energy usage per household (hh) per year (1,544 kWh/hh) and then multiplying by the number of households in question. The only exception was calculating the low cost of diesel, where 3,066 kWh/hh was used for the expected energy usage; a limiting assumption of diesel being used 24/7 to meet a constant load.

References

1. [dataset]* Erik Brown, Feasibility of isg-pv system for off-grid communities (Dryad https://doi.org/10.5061/dryad.rn8pk0pc9) (2022).
2. S Pfenninger, I Staffell, Long-term patterns of european pv output using 30 years of validated hourly reanalysis and satellite data. *Energy* **114**, 1251–1256 (2016).
3. S Chaudhari, Y Pokhrel, E Moran, G Miguez-Macho, Multi-decadal hydrologic change and variability in the amazon river basin: understanding the terrestrial water storage variations and drought characteristics. *Hydrol. Earth Syst. Sci.* **23**, 2841–2862 (2019).
4. T Huld, R Gottschalg, HG Beyer, M Topic, Mapping the performance of pv modules, effects of module type and data averaging. *Sol. Energy* **84**, 324–338 (2010).
5. SmartHydro, Smart prices and products overview (https://www.smart-hydro.de/renewable-energy-systems/prices-hydrokinetic-photovoltaic/, Last Checked: January 17, 2022) (2019).
6. LS Lasdon, AD Waren, A Jain, MW Ratner, Design and testing of a generalized reduced gradient code for nonlinear programming. *ACM Transactions on Math. Softw.* **4**, 34–50 (1978).
7. LS Lasdon, RL Fox, MW Ratner, Nonlinear optimization using the generalized reduced gradient method, (Office of Naval Research), Technical Report AD0774723 (1973).
8. Fundación Proteger, International Rivers, and ECOA, Dams in amazonia (WayBackMachine: https://web.archive.org/web/20181230133801/https://dams-info.org/en, Last Checked: January 17, 2022) (2018).
9. ANEEL, Relatórios de consumo e receita de distribuição (https://www.aneel.gov.br/relatorios-de-consumo-e-receita, Last Checked: June 9, 2020) (2019).
10. ESMAP, Reducing the cost of grid extension for rural electrication, (NRECA International, Ltd.), Technical Report ESM 227 (2000).
11. ONS, Par/pel executivo 2020-2024, (ONS), Technical Report http://www.ons.org.br/AcervoDigitalDocumentosEPublicacoes/REVISTA%20PAR%202020-2024_Final_Online%20(1).pdf (2019).
12. ONS, Par/pel executivo 2019-2023, (ONS), Technical Report http://www.ons.org.br/AcervoDigitalDocumentosEPublicacoes/PAR2019_2023_sumario_executivo.pdf (2018).
13. ONS, Par 2017-2019 plano de ampliações e reforços nas instalações de transmissão do sin, (ONS), Technical Report http://www.ons.org.br/AcervoDigitalDocumentosEPublicacoes/PAR2017-2019_sumario_executivo.PDF (2016).
14. A Sanoh, L Parshall, OF Sarr, S Kum, V Modi, Local and national electricity planning in senegal: Scenarios and policies. *Energy for Sustain. Dev.* **16**, 13–25 (2012).
15. L Parshall, D Pillai, S Mohan, A Sanoh, V Modib, National electricity planning in settings with low pre-existing grid coverage:development of a spatial model and case study of kenya. *Energy Policy* **37**, 2395–2410 (2009).
16. U Deichmann, C Meisner, S Murray, D Wheeler, The economics of renewable energy expansion in rural sub-saharan africa. *Energy Policy* **39**, 215–227 (2011).
17. T Levin, VM Thomas, Can developing countries leapfrog the centralized electrification paradigm? *Energy for Sustain. Dev.* **31**, 97–107 (2016).
18. C Petheram, T McMahon, Dams, dam costs and damnable overruns. *J. Hydrol. X* **3** (2019).
19. A Ansar, B Flyvbjerg, A Budzier, D Lunn, Should we build more large dams? the actual costs of 477 hydropower megaproject development. *Energy Policy* **69**, 43–56 (2014).
20. IEA/NEA, Projected costs of generating electricity 2010 edition, (IEA/NEA), Technical Report https://www.oecd-nea.org/jcms/pl_14482/projected-costs-of-generating-electricity-2010-edition?details=true (2010).
21. MC Quintas, CJC Blanco, ALA Mesquita, Analysis of two schemes using micro hydroelectric power (mhps) in the amazon with environmental sustainability and energy and economic feasibility. *Environ. Dev. Sustain.* **14**, 283–295 (2012).
22. W Bank, Pump price for diesel fuel usd per liter (https://data.worldbank.org/indicator/EP.PMP.DESL.CD?locations=BR, Last Checked: May 19, 2020) (2016).
